# The Ser19Stop single nucleotide polymorphism (SNP) of human PHYHIPL affects the cerebellum in mice

**DOI:** 10.1186/s13041-021-00766-x

**Published:** 2021-03-12

**Authors:** Hisako Sugimoto, Takuro Horii, Jun-Na Hirota, Yoshitake Sano, Yo Shinoda, Ayumu Konno, Hirokazu Hirai, Yasuki Ishizaki, Hajime Hirase, Izuho Hatada, Teiichi Furuichi, Tetsushi Sadakata

**Affiliations:** 1 Education and Research Support Center, Gunma University Graduate School of Medicine, 3-39-22 Showa-machi, Maebashi, Gunma 371-8511 Japan; 2 Laboratory of Genome Science, Biosignal Genome Resource Center, Institute for Molecular and Cellular Regulation, Gunma University, 3-39-15 Showa-machi, Maebashi, 371-8512 Japan; 3 Department of Applied Biological Science, Faculty of Science and Technology, Tokyo University of Science, 2641 Yamazaki, Noda, Chiba 278-8510 Japan; 4 Department of Environmental Health, School of Pharmacy, Tokyo University of Pharmacy and Life Sciences, 1432-1 Horinouchi, Hachioji, Tokyo 192-0392 Japan; 5 Department of Neurophysiology and Neural Repair, Gunma University Graduate School of Medicine, Maebashi, Gunma 371-8511 Japan; 6 Department of Molecular and Cellular Neurobiology, Gunma University Graduate School of Medicine, 3-39-22 Showa-machi, Maebashi, Gunma 371-8511 Japan; 7 Center for Translational Neuromedicine, Faculty of Medical and Health Sciences, University of Copenhagen, Blegdamsvej 3B, 2200 Copenhagen N, Denmark

**Keywords:** PHYHIPL, PHYHIP, dbSNP, HapMap Project, Cerebellum, Purkinje cell

## Abstract

The HapMap Project is a major international research effort to construct a resource to facilitate the discovery of relationships between human genetic variations and health and disease. The Ser19Stop single nucleotide polymorphism (SNP) of human phytanoyl-CoA hydroxylase-interacting protein-like (PHYHIPL) gene was detected in HapMap project and registered in the dbSNP. PHYHIPL gene expression is altered in global ischemia and glioblastoma multiforme. However, the function of PHYHIPL is unknown. We generated PHYHIPL Ser19Stop knock-in mice and found that PHYHIPL impacts the morphology of cerebellar Purkinje cells (PCs), the innervation of climbing fibers to PCs, the inhibitory inputs to PCs from molecular layer interneurons, and motor learning ability. Thus, the Ser19Stop SNP of the PHYHIPL gene may be associated with cerebellum-related diseases.

**Supplementary Information:**

The online version contains supplementary material available at 10.1186/s13041-021-00766-x.

## Introduction

PHYHIP-like (PHYHIPL) is a paralog of the phytanoyl-CoA hydroxylase-interacting protein (PHYHIP). Hereafter, we abbreviate PHYHIPL as PHY2 and the original PHYHIP as PHY1. Kim and colleagues first cloned PHY1, which showed a brain-specific expression pattern [1]. They also reported that PHY1 interacts with the Refsum disease gene product phytanoyl-CoA alpha-hydroxylase (PAHX) [1] and brain-specific angiogenesis inhibitor 1 (BAI1) [2]. Another group reported that PHY1 interacts with a dual-specificity tyrosine-phosphorylated and regulated kinase 1A (DYRK1A), which is located in a Down syndrome critical region [3]. Besides, visual stimulation is essential to maintain PHY1 expression in the retina [4]. On the other hand, the gene expression of PHY2 is altered in global ischemia [5] and glioblastoma multiforme [6], but its function remains unknown.

The International HapMap Consortium has developed the HapMap, a resource that describes the common patterns of human genetic variation. HapMap data are deposited in the Single-Nucleotide Polymorphism database (dbSNP), a variation database at the National Center for Biotechnology Information (NCBI). The Ser19Stop SNP of the human PHY2 gene was identified by the HapMap project and registered in the dbSNP (Database ID: rs7907875). The frequency of the Ser19Stop SNP of human PHY2 in the Japanese population is high (2.3%). However, all Ser19Stop SNP are registered as heterozygote and the HapMap project identified no homozygote. There is a 0.05% chance of finding heterozygotes in the Japanese population. Homozygotes may suffer from some disease, which would explain why they have been omitted from the collection of DNA. Therefore, we generated homozygous Ser19Stop PHY2 knock-in mice and analyzed their phenotypes to examine the association between some human diseases and the Ser19Stop SNP.

## Results and discussion

**Fig. 1:**
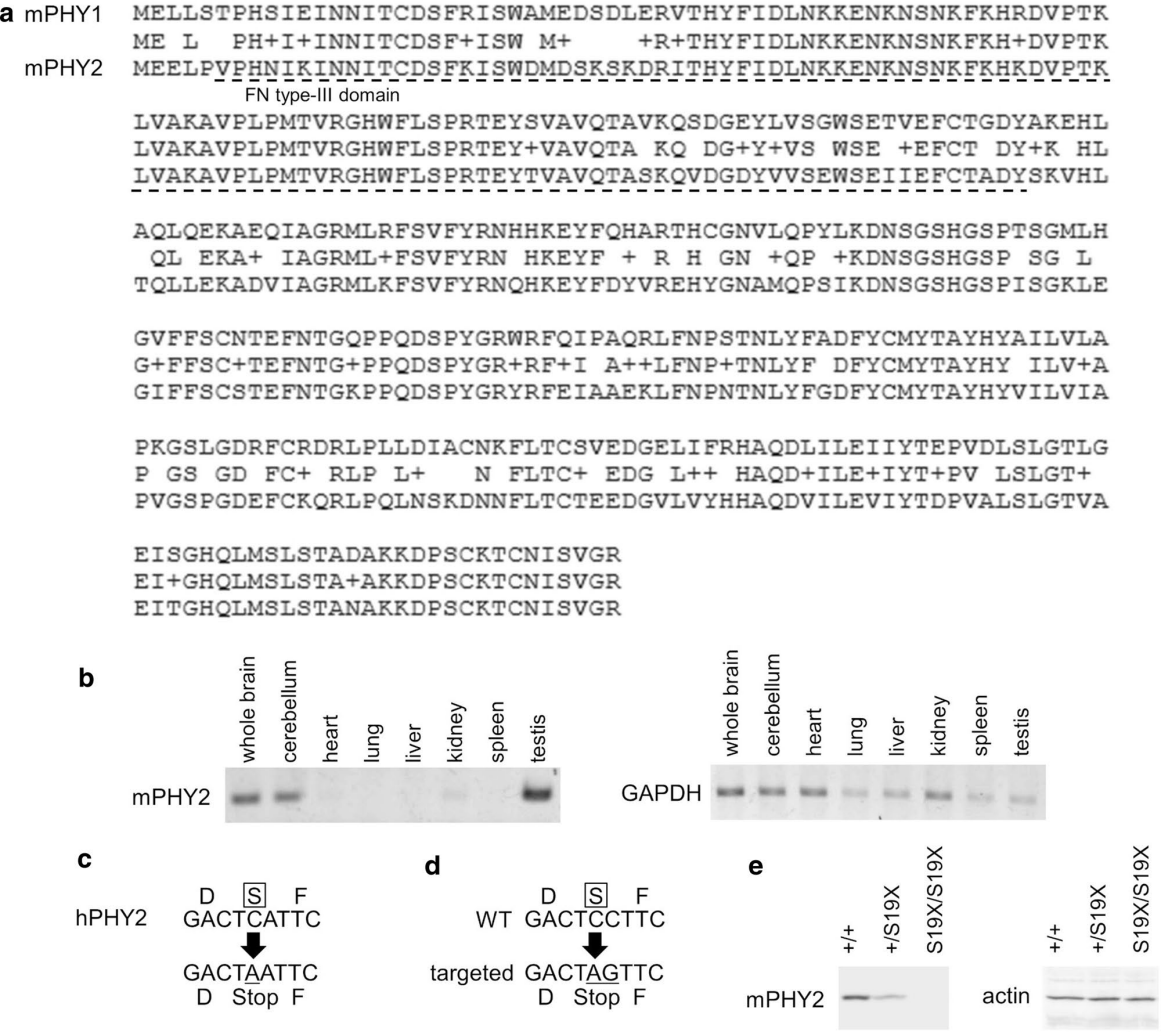
Generation of PHY2 Ser19Stop knock-in mice. **a** Alignment of mouse PHY1 (top) and PHY2 (bottom) protein sequences. Identical amino acids are shown in the middle. The dashed line represents the Fibronectin type-III domain. **b** Tissue distribution of PHY2 expression in P56 mice. Glyceraldehyde-3-phosphate dehydrogenase (GAPDH) was used as a control. **c** The Ser19Stop SNP of human PHY2 registered in the dbSNP (rs7907875). **d** A portion of the coding region in the mouse PHY2 gene was replaced with Ser19Stop SNP. **e** Immunoblot analysis of P21 cerebellum of wild-type (PHY2^+/+^), heterozygous (PHY2^+/S19X^), and homozygous (PHY2^S19X/S19X^) mice. Protein lysates were immunoblotted with anti-PHY2 and anti-actin antibodies

The mouse PHY2 and PHY1 proteins share 75% amino-acid sequence identity (Fig. 1a). PHY2 has structural features similar to the Fibronectin type-III domain, which may mediate specific protein–protein interactions in both intracellular and extracellular compartments (Fig. 1a). The RT-PCR analysis showed a high PHY2 mRNA expression in the brain and testis (Fig. 1b), indicating that PHY1 and PHY2 have a similar tissue distribution [1].

The Ser19Stop SNP of human PHY2 was registered in the dbSNP (Fig. 1c). We found this SNP manually among the SNPs corresponding to the following search criteria: stop gained [function class] AND by frequency [validation status] AND by cluster [validation status] AND Homo sapiens [organism]. The frequency of Ser19Stop in Japanese and American populations is 2.3% and 0.9%, respectively. All Ser19Stop SNPs registered in the HapMap project are heterozygotes. The mouse and human PHY2 are highly homologous and both have a serine in position 19 (Additional File. 1: Fig. S1). Therefore, we inserted the Ser19Stop (S19X) mutation in mouse PHY2 by CRISPR/Cas9-mediated gene knock-in strategy (Fig. 1d, Additional File. 1: Fig. S1). We verified the S19X knock-in by sequencing and Western blotting (Fig. 1e).

**Fig. 2:**
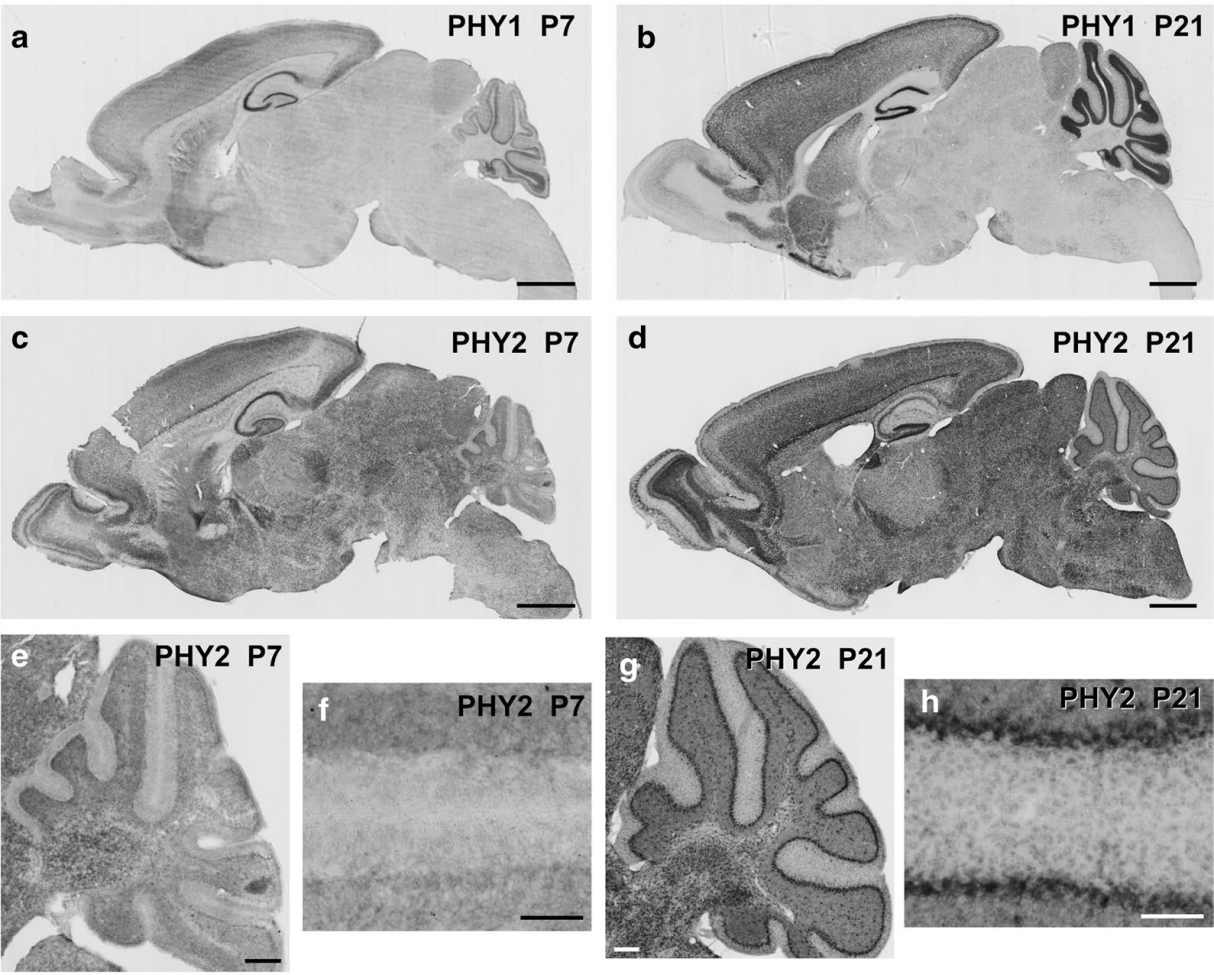
Expression of PHY1 and PHY2 mRNA in mouse brains. **a**, **b** In situ hybridization analysis of PHY1 mRNA distribution in P7 (**a**) and P21 (**b**) sagittal brain sections. Scale bars: 1 mm. **c**, **d** In situ hybridization analysis of PHY2 mRNA distribution in P7 (**c**) and P21 (**d**) sagittal brain sections. Scale bars: 1 mm. **e** In situ hybridization analysis of PHY2 mRNA distribution in P7 sagittal brain sections in the cerebellum. Scale bars: 200 µm. **f** In situ hybridization analysis of PHY2 mRNA distribution in P7 sagittal brain sections in the cerebellar cortex. Scale bars: 100 µm. **g** In situ hybridization analysis of PHY2 mRNA distribution in P21 sagittal brain sections in the cerebellum. Scale bars: 200 µm. **h** In situ hybridization analysis of PHY2 mRNA distribution in P21 sagittal brain sections in the cerebellar cortex. Scale bars: 100 µm

The expression of PHY1 is brain-specific [1]. In situ hybridization analyses showed that cerebellar granule cells, hippocampal neurons, and cerebral neurons strongly expressed PHY1 mRNA as previously reported (Fig. 2a, b) [1]. Moreover, we detected low levels of PHY1 mRNA in the striatum and nucleus accumbens (Fig. 2b).

On the contrary, PHY2 was distributed over the whole brain area (Fig. 2c, d). The hippocampal granule and pyramidal cells (Fig. 2c, d), neocortical cells located in the deep layers (Fig. 2c, d), and cerebellar Purkinje cells (Fig. 2g, h) highly expressed PHY2 mRNA. In the cerebellum, the stellate and Golgi cells also weakly expressed PHY2 mRNA (Fig. 2h).

**Fig. 3:**
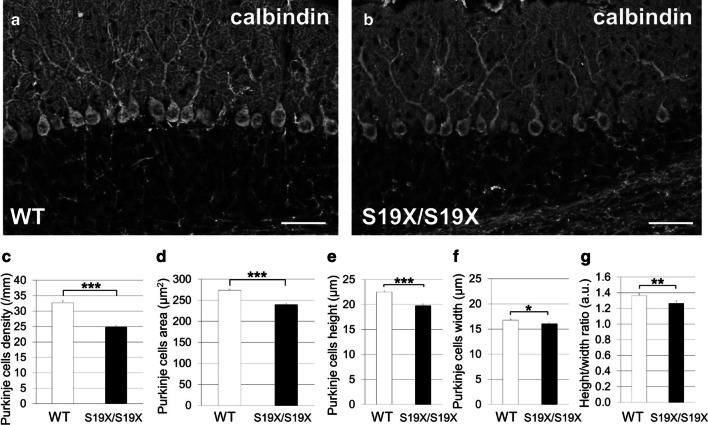
Abnormal PC morphology in PHY2^S19X/S19X^ cerebellum. **a**, **b** Sagittal sections of P21 wild-type (WT) (**a**) and PHY2^S19X/S19X^ (S19X/S19X) (**b**) mouse cerebella immunolabeled with an anti-calbindin antibody. Scale bars: 50 µm. **c** Cell densities of calbindin-positive PCs for WT (white, *n* = 40) and PHY2^S19X/S19X^ (black, *n* = 71) mice at P21. **d** PC soma size for WT (white, *n* = 315) and PHY2^S19X/S19X^ (black, *n* = 289) mice at P21. **e** Longitudinal diameter (height) of the PC soma for WT (white, *n* = 124) and PHY2^S19X/S19X^ (black, *n* = 106) mice at P21. **f** Transversal diameter (width) of the PC soma for WT (white, *n* = 124) and PHY2^S19X/S19X^ (black, *n* = 106) mice at P21. *P* = 0.0314, Student’s *t* test. **g** Height/width ratio of the PC soma for WT (white, *n* = 124) and PHY2^S19X/S19X^ (black, *n* = 106) mice at P21. *P* = 0.00424, Student’s *t* test. The error bars indicate the s.e.m. *P < 0.05; **P < 0.01; ***P < 0.001, Student’s *t* test

Apart from the cerebellum, the brain structure and cytoarchitecture of the PHY2 mutant homozygotes (PHY2^S19X/S19X^) were indistinguishable from those seen in wild-type littermates. In the cerebellum, the PHY2^S19X/S19X^ Purkinje cells (PCs) had significantly lower cell density than wild-type PCs (Fig. 3a–c). Besides, the PHY2^S19X/S19X^ PC soma was smaller than that of wild-type (Fig. 3d). PCs are pear-shaped neurons and their longitudinal diameter is frequently bigger than the transversal diameter (Fig. 3a). Interestingly, the PHY2^S19X/S19X^ PCs were round-shaped and their soma had a smaller height/width ratio than that of wild-type PC soma (Fig. 3e–g). Postmortem neuropathology studies report that a majority of cerebellar specimens from persons diagnosed with autism spectrum disorders have fewer and smaller PCs [7]. Having few and small PCs may reduce the directional coherence and detection of streamlines connecting the cerebellar cortex with the dentate nucleus [8].

**Fig. 4:**
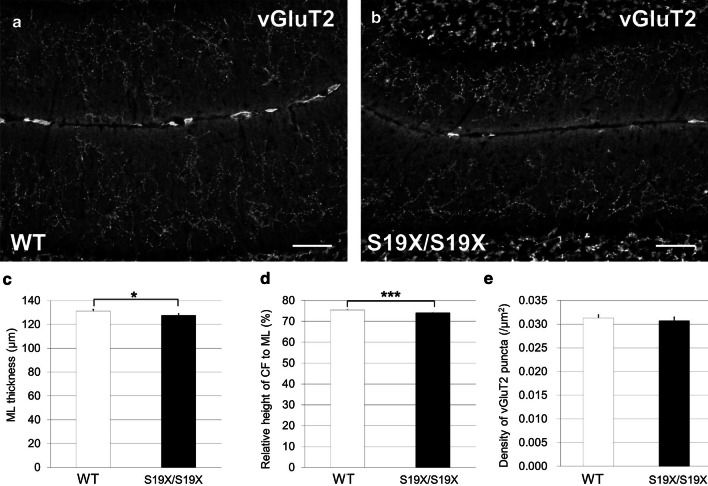
Decreased CF height relative to ML in the PHY2^S19X/S19X^ cerebellum. **a**, **b** Sagittal sections of P21 WT (**a**) and PHY2^S19X/S19X^ (**b**) mice cerebella immunolabeled with an anti-vGluT2 antibody. Scale bars: 50 µm. **c** ML thickness in WT (white, *n* = 340) and PHY2^S19X/S19X^ (black, *n* = 340) mice at P21. *P* = 0.0493, Student’s *t* test. **d** CF height relative to ML in WT (white, *n* = 340) and PHY2^S19X/S19X^ (black, *n* = 340) mice at P21. **e** Density of vGluT2 puncta in the ML of WT (white, *n* = 340) and PHY2^S19X/S19X^ (black, *n* = 340) mice at P21. The error bars indicate the s.e.m. *P < 0.05; ***P < 0.001, Student’s *t* test

In the developing cerebellum, climbing fibers (CFs) surround the basal part of monolayered PC somata and establish synaptic contacts with perisomatic protrusions and thorns. Perisomatic CF synapses then progressively translocate to growing PC dendrites between postnatal week one to three in the mouse cerebellum [9]. When the number of perisomatic CF synapses decreases, the height of the CF projection in the molecular layer (ML) dramatically increases. Dendritic translocation of CFs must be an activity‐dependent process, as administering tetrodotoxin or AMPA receptor blockers atrophy CF innervation in adult rats and mice [10–12]. The dendritic translocation is evaluated as the height of CF projection relative to the thickness of the ML [9]. The thickness of the ML was slightly smaller in the PHY2^S19X/S19X^ mouse cerebellum than in the wild-type cerebellum (Fig. 4a–c). Moreover, the relative height of CF to ML was smaller in the PHY2^S19X/S19X^ cerebellum than in the wild-type cerebellum (Fig. 4d). The wild-type and PHY2^S19X/S19X^ cerebellum had the same vGluT2 puncta density (Fig. 4e). These results indicate that decreased PC activity occurs in the PHY2^S19X/S19X^ mouse cerebellum.

**Fig. 5:**
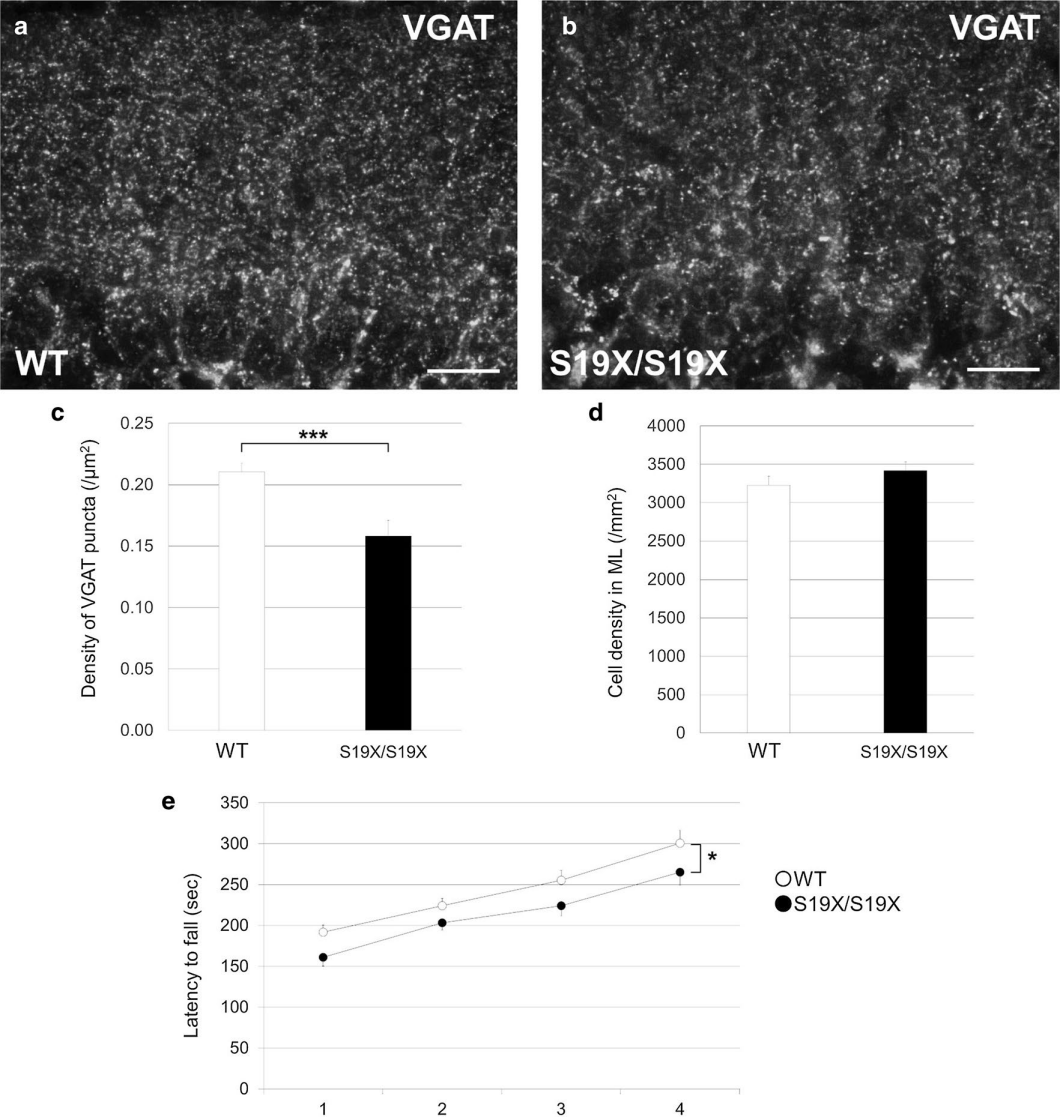
Decreased density of VGAT puncta in the ML of the PHY2^S19X/S19X^ cerebellum and impairment of motor coordination in PHY2^S19X/S19X^ mice. **a**, **b** Sagittal sections of P21 WT (**a**) and PHY2^S19X/S19X^ (**b**) mice cerebella immunolabeled with an anti-VGAT antibody. Scale bars: 20 µm. **c** Density of VGAT puncta in the ML of WT (white, *n* = 31) and PHY2^S19X/S19X^ (black, *n* = 30) mice at P21. **d** Density of DAPI positive cells in the ML of WT (white, *n* = 38) and PHY2^S19X/S19X^ (black, *n* = 48) mice at P21. **e** Fall latency in the rotarod test for WT (white, *n* = 11) and PHY2^S19X/S19X^ (black, *n* = 8) mice at postnatal week 10–11. Data for the 4 days of evaluation is shown. *P = 0.0334, repeated measures ANOVA. The error bars indicate the s.e.m. ***P < 0.001, Student’s *t* test. *P < 0.05, repeated measures ANOVA

PCs receive two excitatory inputs, from parallel fibers (axons of the granule cells) and climbing fibers. They receive inhibitory inputs from two groups of ML interneurons, the basket cells and stellate cells. The spatiotemporal patterns of PC action potentials, which are triggered by the excitatory inputs, are fine‐tuned by the inhibitory inputs from ML interneurons [13, 14]. To clarify the effects of PHY2 on the formation of ML interneuron–PC synapses, we first evaluated the density of VGAT‐positive inhibitory terminals. PHY2^S19X/S19X^ ML had a smaller density of VGAT puncta than wild-type ML (Fig. 5a–c). The cell density in PHY2^S19X/S19X^ and wild-type ML was the same (Fig. 5d). The decrease in VGAT puncta in Fig. 5 may be due to homeostatic plasticity, a process by which neurons adapt to the overall network activity to maintain their firing rates [15]. In dissociated primary cultures of rat neocortex, decreased neuronal activity leads to a decrease in VGAT [16]. Similarly, decreased activity of Purkinje cells may lead to a decrease in VGAT expression in surrounding interneurons due to homeostatic plasticity (Fig. 5c). To conclude the association between our immunohistochemical results and Purkinje cell activity, the physiological activity of Purkinje cells in the PHY2^S19X/S19X^ cerebellum remains to be elucidated.

Since, in home cages, the PHY2^S19X/S19X^ mice had a behavior indistinguishable from that of their wild-type littermates, we analyzed their motor ability more carefully using a rotarod apparatus. Mice of both genotypes learned the task, and the time on the rod gradually increased (Fig. 5e). We monitored the motor learning ability during the test periods. The motor coordination of PHY2^S19X/S19X^ mice was significantly poorer on the fourth testing day [*F*(1,51) = 4.70, *P* = 0.0334]. These results suggest that PHY2^S19X/S19X^ mice have impaired motor coordination.

**Fig. 6:**
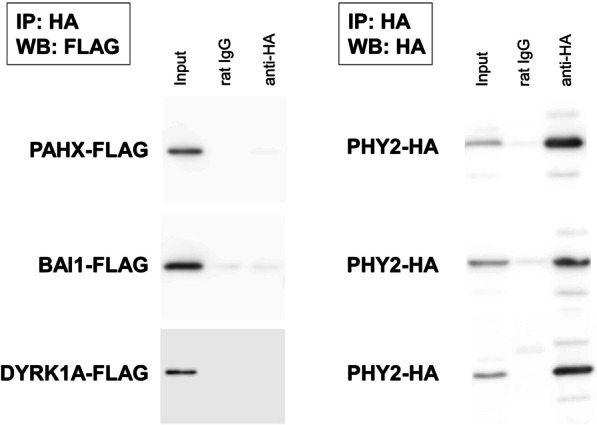
PHY2 interacts with neither PAHX, BAI1, nor DYRK1A. Western blot of PHY2-HA constructs and PAHX-FLAG, BAI1-FLAG, or DYRK1A-FLAG constructs coexpressed in COS-7 cells, coimmunoprecipitated with anti-HA antibody and immunobloted with ant-FLAG and anti-HA antibodies

PHY1 interacts with the Refsum disease gene product PAHX [1], BAI1 [2], and DYRK1A [3]. Therefore, to examine the protein function of PHY2, we measured its binding activity to these three candidates. Figure 6 shows that that none of them bound to PHY2.

The Ser19Stop mouse had morphologically altered cerebellar PCs, abnormal projection of CFs to PC dendrites, decreased formation of ML interneuron-PC synapses, and impaired motor coordination. These results show that Ser19Stop SNP may be associated with cerebellum-related diseases. Thus far, we could not elucidate the PHY2 protein function. We used the Prosite domain database to identify the fibronectin type III domain in PHY2 [17]. The fibronectin type III superfamily has an Ig-like β-sandwich fold [18]. The fibronectin type III domain is a homophilic binding site involved in fibronectin matrix assembly. The interaction between type III and type I domains is critical for the homophilic binding of fibronectin [19]. Therefore, PHY2 may interact with other proteins through its fibronectin type III domain and have distinct functions from PHY1. Thus far, PHY2 binding proteins have been identified by recombinant PHY2 expression in some neuronal cell lines (data not shown). The association between PHY2 SNPs and human diseases remains unelucidated and requires detailed analyses.

## Materials and methods

### Generation of PHYHIPL Ser19Stop knock-in mice

We generated the PHYHIPL Ser19Stop knock-in mice using the electroporation method according to our previous report with some modifications [20]. In this study, we used synthetic crRNA (Alt-R® CRISPR-Cas9 crRNA; IDT, Coralville, IA, USA) and tracrRNA (Alt-R® CRISPR-Cas9 tracrRNA, IDT) instead of single guide RNA (sgRNA). We also designed the corresponding donor single-stranded oligodeoxynucleotides (ssODNs) with 5′- and 3′-homology arms. Target sequence: CATATCCCATGAGATCTTGAAGG. Equal volumes of crRNA and tracrRNA were combined in a duplex buffer (IDT) in a thermal cycler at 95 °C for 5 min following the manufacturer’s protocol. crRNA/tracrRNA (3 μM), recombinant Cas9 protein (100 ng/μL; GeneArt Platinum™ Cas9 Nuclease, Thermo Fisher Scientific, Waltham, MA, USA) and ssODN (400 ng/μL; Ultramer®, IDT) were mixed in Opti-MEM I (Thermo Fisher Scientific) before electroporation. Fertilized eggs were isolated from the superovulated C57BL/6 J female mice 21 h after administering human chorionic gonadotropin (hCG). The electroporation was conducted at the 1-cell stage (24–26 h after hCG), and the 2-cell stage embryos were transferred to the oviduct of pseudopregnant ICR females (Charles River Japan, Yokohama, Japan). We confirmed the mutant alleles of the obtained mice (Fig. 1d) by sequencing PCR products.

### RT-PCR

We produced a series of first-strand cDNAs by reverse-transcription (RT) from the mice total RNA of eight tissue types (brain, cerebellum, heart, lung, liver, kidney, spleen, and testis) using an oligo-dT primer as previously described [21, 22]. We used the following primers: Mouse PHY2 exon 3 forward primer: 5′-GTCGTGTCTGAATGGAGTGAGATTATAGAATTCTG-3′; mouse PHY2 exon 5 reverse primer: 5′-ATCCTGGGGTGGCTTCCCTGTATTG-3′ (product: 297 bp); mouse glyceraldehyde-3-phosphate dehydrogenase (GAPDH) forward primer: 5′-GCCATCAACGACCCCTTCATTGACCTC-3′; mouse GAPDH reverse primer: 5′-GCCATGTAGGCCATGAGGTCCACCAC-3'.

### In situ hybridization (ISH)

We performed the ISH as described previously, with minor modifications [23, 24]. C57BL/6 J male mice were anesthetized deeply with a combination of midazolam, medetomidine and butorphanol tartrate and transcardially perfused with 4% paraformaldehyde (PFA), harvested, post-fixed with 4% PFA at 4 °C for 1 d, and equilibrated in 30% (w/v) sucrose in phosphate-buffered saline (PBS). Sagittal sections (50 μm thickness) were prepared using a cryostat. All steps were performed at room temperature unless indicated otherwise. Sections were incubated with methanol (MeOH) for 2 h, then washed three times for 10 min in PBS containing 0.1% Tween-20 (PBST). Next, they were incubated with 0.5 μg/ml proteinase K (Sigma-Aldrich, St. Louis, MO, USA) in Proteinase K buffer (0.1 M Tris HCl, pH 8.0, 50 mM EDTA) for 30 min at 37 °C, followed by incubation with 0.25% Acetic anhydride in 0.1 M triethanolamine, pH 7.0 for 10 min. Finally, they were washed twice for 5 min in PBST and incubated with hybridization buffer (5 × SSC, 50% formamide, 0.1% Tween-20, 5 × Denhardt’s solution) for 1 h at 60 °C. Before hybridization, digoxigenin (DIG)-labeled cRNA probes in hybridization buffer were denatured at 80 °C for 5 min and then quickly cooled on ice for 10 min. cRNA probes were generated using a DIG RNA labeling kit (Roche, Mannheim, Germany). Hybridization was performed at 60 °C overnight. Sections were washed in 2 × SSC containing 50% formamide and 0.1% Tween-20 (SSCT) twice for 20 min and incubated with 20 µg/ml RNase (Nippon Gene, Tokyo, Japan) in RNase buffer (10 mM Tris–HCl, pH 8.0, 1 mM EDTA, 0.5 M NaCl) for 30 min at 37 °C. They were then washed twice with 2 × SSCT for 15 min at 37 °C and twice with 0.2 × SSCT for 15 min at 37 °C. Then, the sections were incubated with 1% blocking reagent (Roche; 10 mM maleic acid, 15 mM NaCl, pH 7.5) for 1 h, and finally incubated with alkaline phosphatase-conjugated anti-DIG antibody (1:2000, Roche) in blocking reagent at 4 °C overnight. The sections were washed three times with TNT (0.1 M Tris–HCl, pH 7.5, 0.15 M NaCl, 0.05% Tween-20) for 15 min. For staining with nitroblue tetrazolium chloride/5-bromo-4-chloro-3-indolyl phosphate 4-toluidine salt (NBT/BCIP), the signal was developed in 2% (v/v) NBT/BCIP stock solution (Roche) diluted in TNM (0.1 M Tris pH 9.5, 0.1 M NaCl, 10 mM MgCl_2_) at room temperature overnight. Sections were imaged using a NanoZoomer Digital Pathology virtual slide scanner (Hamamatsu Photonics, Hamamatsu, Japan).

### Antibodies

Rat polyclonal anti-PHY2 antibody was raised against the GST-tagged PHY2 (aa 231-297) that was bacterially expressed, and were affinity-purified against the MBP-tagged antigenic proteins that were covalently coupled to CNBr-activated Sepharose 4B.

Rat anti-PHY2 (1 ng/μl), rabbit anti-actin (1:800 dilution; Cat No: A5060; Sigma-Aldrich) mouse monoclonal anti-FLAG (1:1,000 dilution; Cat No: F1804; Sigma-Aldrich) and rat monoclonal anti-HA (1:1,000 dilution; Cat No: 1867423; Roche) were used for Western blotting.

The following primary antibodies were used for immunohistochemistry: mouse monoclonal anti-calbindin (1:500 dilution; Cat No: 214011; Synaptic Systems, Goettingen, Germany), mouse monoclonal anti-vGluT2 (1:300 dilution; Cat No: MAB5504; Millipore, Billerica, MA, USA), and rabbit anti-VGAT (1:500 dilution; Cat No: AB5062P; Millipore) antibodies.

### Immunohistochemistry

We performed immunohistochemistry as described previously [25]. C57BL/6 J male mice were anesthetized deeply with a combination of midazolam, medetomidine and butorphanol tartrate and transcardially perfused with PBS and then with Zamboni's fixative (2% paraformaldehyde in 0.1 M phosphate buffer, pH 7.4, containing 0.2% picric acid). Tissues were dissected, post-fixed in Zamboni's fixative at 4 °C for 5 h and cryoprotected by immersion in 15% sucrose in PBS overnight at 4 °C. After embedding in Tissue-Tek OCT compound (Sakura Finetek, Tokyo, Japan), tissues were frozen and sectioned at a thickness of 15 µm using a cryostat (CM1950, Leica Microsystems, Frankfurt, Germany) at − 18 °C. The sections were air-dried for 1 h and rinsed in PBS three times. After blocking with 5% bovine serum albumin (BSA) and 0.3% Triton X-100 in PBS at room temperature for 1 h, the sections were incubated at 4 °C overnight with the primary antibodies in immunoreaction buffer (2 × PBS containing 0.3% Triton X-100 and 1% BSA). The sections were then washed in PBS, incubated at room temperature for 1 h with the appropriate secondary antibodies in immunoreaction buffer, and washed again in PBS. Stained sections were mounted in DAPI Fluoromount-G® mounting medium (SouthernBiotech, Birmingham, AL, USA) and observed under a fluorescence microscope (BX51, Olympus, Tokyo, Japan) equipped with a CCD camera (VB-7000, Keyence, Osaka, Japan).

### Rotarod test

We evaluated the motor control ability of the mice using a rotarod (MK-610A/RKZ; Muromachi Kikai, Tokyo, Japan) as previously described [26, 27]. Briefly, the male mice had to run backwards to maintain their position on top of a rod revolving at 4 rpm. The mice were subjected to four trials on the rod accelerating from 4 to 40 rpm in 5 min. There was a 20 min pause between trials.

### Immunoprecipitation

We performed immunoprecipitation as previously described [28]. Mouse PHY2 cDNA was subcloned in frame in front of the HA epitope tag sequence in pEF-BOS plasmid [29] to create the C-terminally HA-tagged PHY2 construct, pEF-BOS-PHY2-HA. Similarly, mouse PAHX, BAI1, and DYRK1A were subcloned in frame in front of the FLAG epitope tag sequence. Transient transfection was performed with lipofectamine 3000 reagent (Thermo Fisher Scientific). Forty-eight hours after transfection, COS-7 cells were harvested and lysed in 1.3 ml of lysis buffer (50 mM HEPES, pH 7.4, 10% glycerol, 100 mM NaCl, 0.5 mM MgCl_2_, 2 mM EGTA, and 1% TritonX-100) containing a cocktail of protease inhibitors. After preabsorption with protein A-sepharose, the supernatants were divided equally into two tubes and incubated with 0.5 µg normal rat IgG or anti-HA antibody (Cat No: 1867423; Roche), and the immunocomplexes were then associated with protein A-sepharose resins. The resins were washed five times with lysis buffer, and the bound proteins were separated on an SDS-PAGE gel and transferred to a nitrocellulose membrane for analysis with anti-HA or anti-FLAG antibodies.

## Supplementary Information

**Additional file 1: Fig. S1.** Generation of PHY2 Ser19Stop knock-in mice. Alignment of human PHY2 (top) and mouse PHY2 (bottom) protein sequences. Identical amino acids are shown in the middle. The Ser19Stop SNP of human PHY2 registered in the dbSNP (rs7907875) is also shown. The Ser19 is conserved between human and mouse PHY2.

## Data Availability

All data generated or analyzed during this study are included in this published article and its additional information files.

## Abbreviations

PHYHIPL: Phytanoyl-CoA hydroxylase-interacting protein-like
SNP: Single nucleotide polymorphism
PC: Purkinje cell
CF: Climbing fiber
ML: Molecular layer
