# Supplementary figures and images for: The Ser19Stop single nucleotide polymorphism (SNP) of human PHYHIPL affects the cerebellum in mice
Source: Mol Brain. 2021 Mar 12;14:52. doi: 10.1186/s13041-021-00766-x (PMC7953787; doi:10.1186/s13041-021-00766-x)

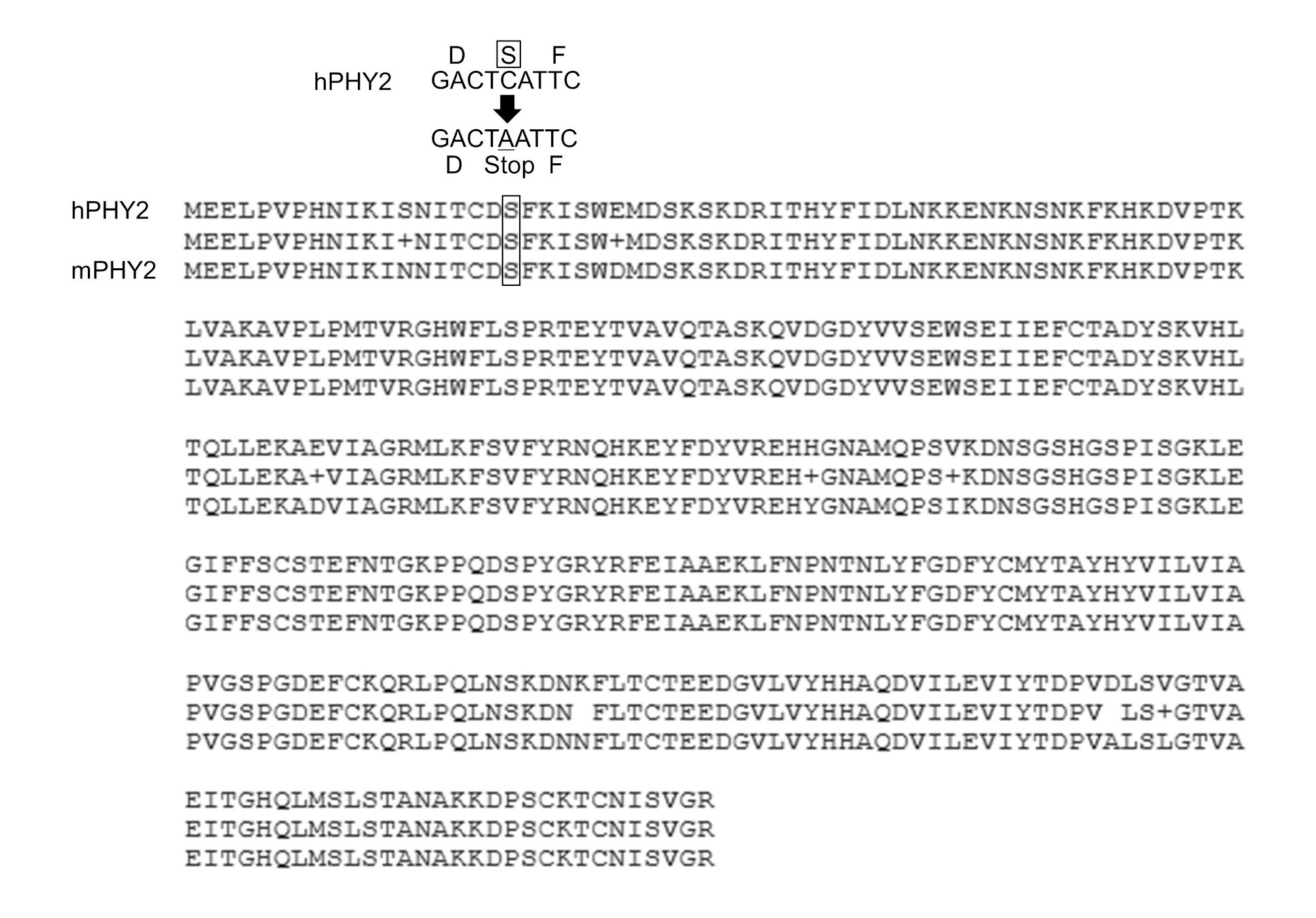

Supplement: Supplementary file 1 — Additional file 1: Fig. S1. Generation of PHY2 Ser19Stop knock-in mice. Alignment of human PHY2 (top) and mouse PHY2 (bottom) protein sequences. Identical amino acids are shown in the middle. The Ser19Stop SNP of human PHY2 registered in the dbSNP (rs7907875) is also shown. The Ser19 is conserved between human and mouse PHY2. [file 13041_2021_766_MOESM1_ESM.jpg]
